# The presence and impact of reference bias on population genomic studies of prehistoric human populations

**DOI:** 10.1371/journal.pgen.1008302

**Published:** 2019-07-26

**Authors:** Torsten Günther, Carl Nettelblad

**Affiliations:** 1 Human Evolution, Department of Organismal Biology, Uppsala University, Uppsala, Sweden; 2 Division of Scientific Computing, Department of Information Technology, Science for Life Laboratory, Uppsala University, Uppsala, Sweden; University of Chicago, UNITED STATES

## Abstract

Haploid high quality reference genomes are an important resource in genomic research projects. A consequence is that DNA fragments carrying the reference allele will be more likely to map successfully, or receive higher quality scores. This reference bias can have effects on downstream population genomic analysis when heterozygous sites are falsely considered homozygous for the reference allele. In palaeogenomic studies of human populations, mapping against the human reference genome is used to identify endogenous human sequences. Ancient DNA studies usually operate with low sequencing coverages and fragmentation of DNA molecules causes a large proportion of the sequenced fragments to be shorter than 50 bp—reducing the amount of accepted mismatches, and increasing the probability of multiple matching sites in the genome. These ancient DNA specific properties are potentially exacerbating the impact of reference bias on downstream analyses, especially since most studies of ancient human populations use pseudo-haploid data, i.e. they randomly sample only one sequencing read per site. We show that reference bias is pervasive in published ancient DNA sequence data of prehistoric humans with some differences between individual genomic regions. We illustrate that the strength of reference bias is negatively correlated with fragment length. Most genomic regions we investigated show little to no mapping bias but even a small proportion of sites with bias can impact analyses of those particular loci or slightly skew genome-wide estimates. Therefore, reference bias has the potential to cause minor but significant differences in the results of downstream analyses such as population allele sharing, heterozygosity estimates and estimates of archaic ancestry. These spurious results highlight how important it is to be aware of these technical artifacts and that we need strategies to mitigate the effect. Therefore, we suggest some post-mapping filtering strategies to resolve reference bias which help to reduce its impact substantially.

## Author summary

Mapping next-generation sequencing reads to a single linear reference genomes comes with the inherent problem that alleles not found in the reference sequence will achieve lower mapping scores. This reference bias can cause heterozygous sites to be falsely called as homozygous which will have an effect on downstream analysis of the data. We investigate this issue in published ancient DNA data from human populations and find that reference bias is a pervasive phenomenon across data sets. The effect is exacerbated in paleogenomic data due to the short fragments of authentic ancient DNA and the common practice of using pseudo-haploid data. We show that—depending on the circumstances—reference bias can lead to slightly skewed results in population genetic analyses such as estimates of heterozygosity, studies of population affinities or estimates of archaic ancestry. Finally, we propose filtering strategies to alleviate the impact of reference bias. We make the scripts used for filtering publicly available.

## Introduction

The possibility to sequence whole genomes in a cost-efficient way has revolutionized the way how we do genetic and population genetic research. Annotated, high-quality reference genomes are a cornerstone for resequencing surveys which aim to study the genetic variation and demographic history of an entire species. Resequencing studies usually align the sequences of all studied individuals to a linear haploid reference sequence originating from a single individual or a mosaic of several individuals. In each site, this haploid sequence will only represent a single allele out of the entire genetic variation of the species. An inherent consequence is some degree of bias towards the alleles present in that reference sequence (“reference bias”). Sequencing reads carrying an alternative allele will naturally have mismatches in the alignment to the reference genome and consequently have lower mapping scores than reads carrying the same allele as the reference. This effect increases with genetic distance from the reference genome, which is of particular interest when using a reference genome from a related species for mapping [1–3]. Generally, reference bias can influence variant calling by missing alternative alleles or by wrongly calling heterozygous sites as homozygous for the reference reference allele [4, 5] which is known to influence estimates of heterozygosity and allele frequencies [6–8].

The field of palaeogenomics and the population genomic analysis of DNA obtained from hominin remains has led to a number of important insights and groundbreaking results in recent years, including admixture between different hominin groups, migrations of prehistoric humans and the evolution of different phenotypes [9–14]. DNA preservation poses a major challenge for these studies, as fragmentation causes most authentic sequences to be shorter than 100 bp, and deamination damage increases the number of mismatches and can even mimic genetic variation at transition sites [15–17].

In addition to fragmentation and other post-mortem damages, low coverage data is a major limiting factor for ancient DNA studies. These low coverages do not permit calling diploid genotypes so a very common approach is to use “pseudo-haploid” data: at each known single nucleotide polymorphisms (SNP) site one sequencing read is picked in order to represent a haploid genotype of that individual. The single read is either chosen at random or to represent the most common allele among all reads mapping to the site. This approach would not introduce bias if the reads were a random representation of the chromosomes carried by the individual. Reference bias, however, would introduce some skew towards the reference allele at heterozygous sites. These characteristics of ancient DNA and practices used in palaeogenomic studies make them particularly vulnerable to reference bias [18–20]. It has been shown that pseudo-haploid data can be more biased than imputed genotypes [21], and that reference bias and fragment length artifacts can interfere with phylogenetic classifications [3]. Reference bias can influence downstream analyses if these are based on estimating allele frequencies in a population, or studying pairwise allele sharing between individuals and groups.

This study investigates the presence and impact of reference bias in studies of prehistoric human populations using genomic ancient DNA. We first illustrate its abundance in published data from ancient human and archaic hominins, and illustrate how it is influenced by standard data processing. We then show how reference bias can influence some basic population genetic analyses such as population affinities and heterozygosity. Finally, we discuss some possible data filtering strategies in order to mitigate reference bias in ancient DNA studies.

## Results

### Mapping quality filtering

We first investigate whether reference bias is present in published ancient DNA data. We restrict our analysis to known biallelic SNPs, as most population genomic analyses are using SNPs and the allele frequencies at those positions. In particular, we are only using transversion polymorphisms (to avoid the effect of post-mortem deaminations) and sites identified to be polymorphic in a world-wide set of modern human populations [22]. We investigate supposedly heterozygous sites (defined as sites covered by at least 10 reads with at least 25% representing the minor allele) in a set of published medium to high coverage human and hominin genomes (Table 1). We note that our approach does not include any rescaling of base qualities, as such approaches usually take the reference allele into account which may amplify reference bias.

**Table 1:** Information on the published medium to high coverage palaeogenomic and archaeogenomic data used in this study.

Sample ID	(Partial) UDG treatment^$^	SNP capture	Average sequencing depth± SE^†^	Number of SNPs^†^ depth ≥ 10*x*	Reference
Stuttgart	X		16.0 ± 7.0	916,374	[23]
Loschbour	X		18.2 ± 7.6	950,730	[23]
Ust’-Ishim	X		31.8 ± 8.0	1,016,515	[24]
sf12^‡^	X		66.3 ± 19.5	1,018,604	[25]
baa001^‡^	X		13.7 ± 7.0	807,484	[26]
Kotias^‡^			13.3 ± 7.7	732,775	[27]
Bichon^‡^			15.4 ± 10.2	731,151	[27]
ne1			19.5 ± 7.5	971,184	[28]
br2			19.3 ± 6.1	993,160	[28]
atp016^‡^			14.6 ± 6.4	888,522	[29]
Rathlin1^‡^			11.2 ± 6.1	655,195	[30]
Ballynahatty^‡^			11.2 ± 6.1	655,947	[30]
I0054	X	X	2.9 ± 8.1	73,901	[31]
I0103	X	X	2.8 ± 7.2	79,825	[31]
I0118	X	X	2.0 ± 4.8	70,872	[31]
I0172	X	X	4.0 ± 9.4	94,558	[31]
I0408	X	X	2.0 ± 6.6	58,358	[31]
I0412	X	X	2.2 ± 6.1	64,057	[31]
I0585	X	X	2.9 ± 8.3	63,176	[31]
TAF011^‡^	X	X	1.1 ± 3.4	37,737	[32]
TAF013^‡^	X	X	0.9 ± 3.0	30,390	[32]
TAF014^‡^	X	X	0.8 ± 2.4	22,648	[32]
AltaiNeandertal	X		47.7 ± 12.3	1,019,927	[33]
VindijaNeandertal	X		28.1 ± 9.8	1,009,725	[34]
Denisovan	X		28.5 ± 8.1	1,013,485	[35]

^‡^ Samples for which unmapped reads were obtained

^$^ Enzymatic repair of deamination damages

^†^ at 1,022,984 analyzed SGDP SNPs, using a minimum mapping quality of 30

**Fig 1:**
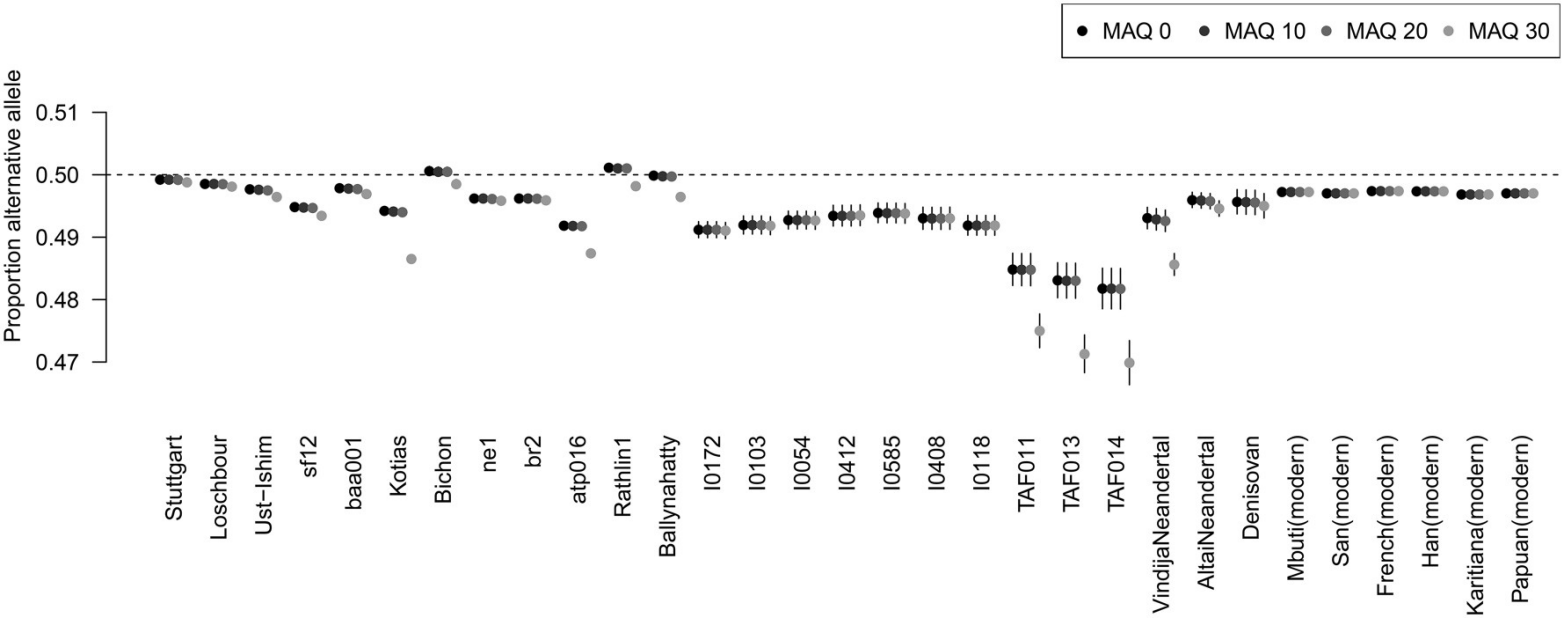
Reference bias in published genome-wide ancient DNA datasets for different minimum mapping quality thresholds. The plot shows the average proportion of reads at heterozygous transversion sites (see Methods) representing the alternative allele. Error bars indicate two standard errors of the mean.

At a heterozygous site, a DNA extract of an individual should contain the same number of reference and alternative fragments. We observe that after mapping to the human reference genome the average proportion of alternative alleles is lower than the expected 50 percent for most of the individuals investigated (Fig 1), regardless of whether they represent libraries with enzymatically repaired post-mortem deamination damage or not (Table 1). Samples for which we used SNP capture data (Table 1) [31, 32] show slightly stronger reference bias than shotgun sequenced samples but they are also characterized by shorter fragments which can influence the strength of reference bias (see below). For comparison, we added six high-coverage modern individuals from diverse continental origin [35] which also show proportions below 50 percent but higher than most ancient individuals highlighting that some degree of mapping bias could be present in NGS data of modern populations as well.

As sequence fragments carrying the alternative allele will show an elevated number of mismatches to the reference genome, mapping quality seems a natural filter to avoid reference bias. Consistent with this expectation, we see a slightly stronger reference bias for stricter mapping quality filters. Lowering the mapping quality cutoff can have other detrimental effects, however, for example an enrichment of microbial contamination [36] or sequences not uniquely mapping to a particular region of the genome. As the qualities of the base calls have not been rescaled after mapping to the reference genome, we do not see an effect of different minimum base quality thresholds on reference bias (S1 Fig).

Post-mortem deamination is a major issue in the analysis of ancient DNA data creating additional mismatches between the sequence reads and the reference genome. We were surprised to see individuals with enzymatic repair of these damages did not systematically perform better in Fig 1 as the expected number of mismatches would be lower for those. To follow up on this, we investigated individual libraries (both damage-repaired and non damage-repaired) of the Vindija Neandertal [34] separately (S2 Fig). All non-damage repaired libraries together show a stronger reference bias than the single damage-repaired library but the latter is also characterized by longer fragment length (see observations below). Furthermore, single non-damage repaired libraries show both higher and lower reference bias without a clear trend which suggests that the influence of post-mortem damage on reference bias is not major.

Investigating pairwise correlations between the proportion of alternative alleles at sites considered heterozygous in both individuals shows significantly positive correlations in most cases (S1 Table). This indicates that the strength of reference (and alternative) bias may differ regionally across the genome, so there could be an effect of sequence context and uniqueness of the specific sequences across the genome. The highest correlations are observed between samples from the same study or produced by the same institute suggesting that similar wet lab techniques also influence this effect.

### Distribution of bias

To investigate the distribution of reference bias instead of just averages as above, we modified original reads to carry opposite alleles at each SNP site and remapped them. We created such a virtual read set for the Scandinavian Mesolithic hunter-gatherer sf12 and the Siberian Ust’-Ishim individual.

In total, 1,022,747 SNPs were identified for sf12, and 1,022,605 for Ust’-Ishim. Out of these, 63.04% and 87.90%, respectively, showed the perfect allelic balance of 0.5 as expected by design from the dataset. The smaller number of balanced SNPs for sf12 is mainly due to increased resolution of twice the number of sequencing reads as a single non-matching read would cause deviations from the perfect 50/50 ratio in this analysis. We only considered reads that map back to their original location from the first mapping round. A very limited number of SNPs were also affected by reads that mapped back with sufficient quality, but to a different genomic location. The proportions of alternative alleles are summarized in Table 2. Notably, there is a subset of SNPs showing alternative as opposed to reference bias. There is also a subset of SNPs where the bias is total, i.e. only one of the two alleles is ever mapped back successfully within this dataset. The distribution across the genome of sites deviating from the balanced case is similar to the overall density of the SNPs used—all chromosomes and chromosomal regions are affected. We also checked the overlap between the two individuals. 1,022,343 SNPs fulfilled the uniqueness filtering conditions and were successfully identified in at least one read in sf12 as well as Ust’-Ishim. Out of these, 584,434 (57.07%) showed perfect allelic balance in both individuals.

**Table 2:** Proportion of alternative alleles when mapping back original reads and virtual opposite allele reads for the sf12 and Ust’-Ishim individuals.

Proportion of alternative alleles	Individual			
	sf12		Ust’-Ishim	
	# SNPs	Percentage	# SNPs	Percentage
Filtered for uniquely mapping SNPs				
0	19	0.00%	24	0.00%
(0, 0.4)	908	0.09%	687	0.07%
[0.4, 0.5)	329896	32.26%	111894	10.94%
0.5	644746	63.04%	898825	87.90%
(0.5, 0.6]	46964	4.59%	11080	1.08%
(0.6, 1)	113	0.01%	88	0.01%
1	11	0.00%	7	0.00%
Unfiltered SNPs				
0	96	0.01%	1228	0.07%
(0, 0.4)	16177	0.96%	27425	1.62%
[0.4, 0.5)	687703	40.64%	282667	16.71%
0.5	912384	53.92%	1359770	80.41%
(0.5, 0.6]	75448	4.46%	19704	1.17%
(0.6, 1)	325	0.02%	286	0.02%
1	36	0.00%	34	0.00%

To investigate further, we also tried 1,693,337 SGDP transversion SNPs without applying the mappability filter. This naturally increased the number of identified SNPs, but at the cost of an even lower proportion of SNPs in perfect allelic balance, and markedly fatter tails in the distribution (0.97% with an allele fraction below 0.4 for sf12, vs. 0.09% with the filtering in place).

### The influence of fragment length

Most mapping strategies set the number of allowed mismatches relative to the length of the sequenced fragment. Therefore, shorter fragments might show a stronger reference bias than long fragments. To investigate this, we used the 57x genome generated for the Scandinavian Mesolithic hunter-gatherer sf12 [25] and partitioned the data into fragment length bins. The large amount of data allows us to still have a sufficient number of SNPs covered at 10x or more for each of the length bins.

**Fig 2:**
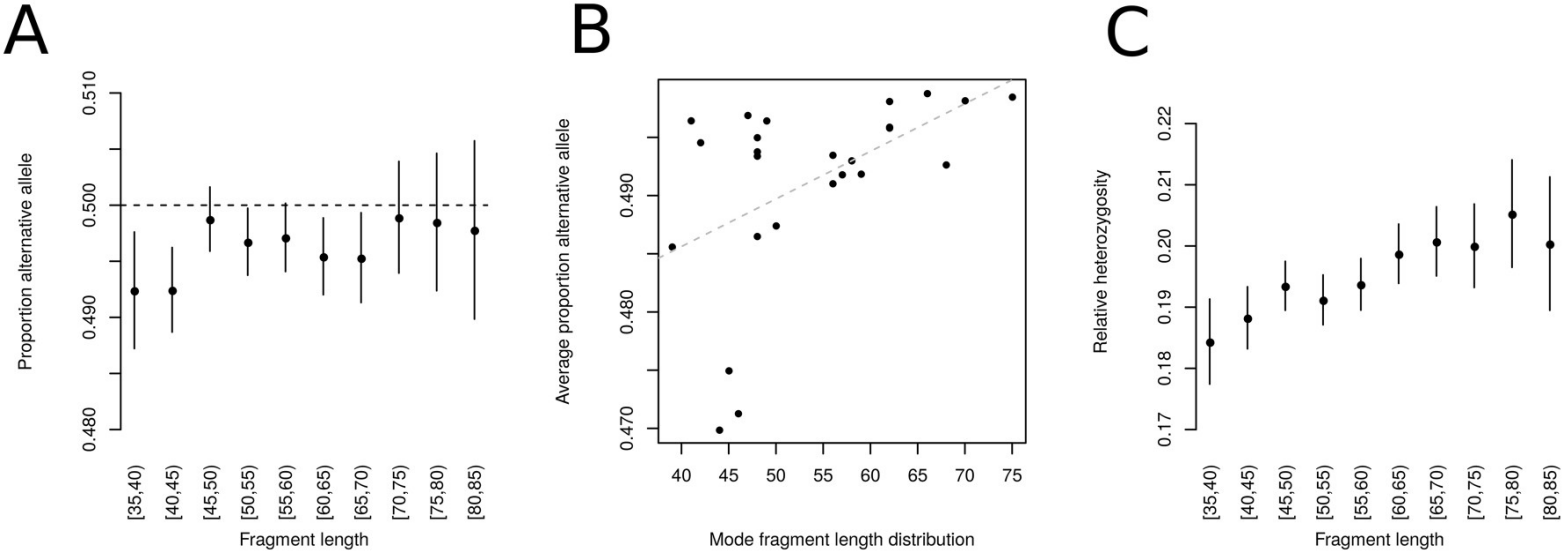
Connection between fragment length and reference bias. (A) Proportion of alternative allele for different fragment length bins in the high coverage individual sf12. (B) Correlation between average proportion of alternative alleles and the mode of the fragment size distribution across all investigated individuals. (C) Proportion of heterozygous sites among all sites with sufficient coverage for different fragment length bins in the high coverage individual sf12. All error bars indicate two standard errors.

Somewhat expectedly, shorter fragments display a stronger reference bias than longer sequences (Fig 2A). Generally, fragment length might be a main driver of reference bias across all samples as the mode of each individual’s fragment size distribution is highly correlated with the average proportion of alternative alleles at heterozygous sites (Pearson’s *r* = 0.496, *p* = 0.0118; Fig 2B). This also has an effect on the proportion of sites considered heterozygous among all sites analyzed which can be seen as a relative measure for the individual’s heterozygosity (Fig 2C). In fact, different fragment length bins of the same individual produce heterozygosity estimates that do not overlap in their 95% confidence interval (Fig 2C). This represents a general limitation for estimating heterozygosity from ancient DNA data which may to some degree explain the generally low diversity estimates for many prehistoric groups [37–39]. The potential of obtaining significantly different estimates for the same population genetic statistic may also have enormous effects on other downstream analyses such as allele sharing and population structure.

### Impact on measures of allele sharing

In order to investigate the influence of reference bias on measures of allele sharing, we calculated different combinations of *D* statistics of the form *D*(*Chimp*, *X*; *Y*, *Z*), where *X* is a modern human population, and *Y* and *Z* are two different treatments of the same individual sf12. Therefore, the expectation for *D* is 0, but differences in reference bias between *Y* and *Z* could lead to spurious allele sharing between population *X* and a deviation from 0. Negative values of *D* indicate more allele sharing of *X* with *Y* while positive values indicate an excess of shared alleles between *X* and *Z*. The populations *X* were grouped by continental origin and we calculated the statistics separately for whole genome shotgun data (SGDP) [22] and populations genotyped using a SNP array (HO) [23].

**Fig 3:**
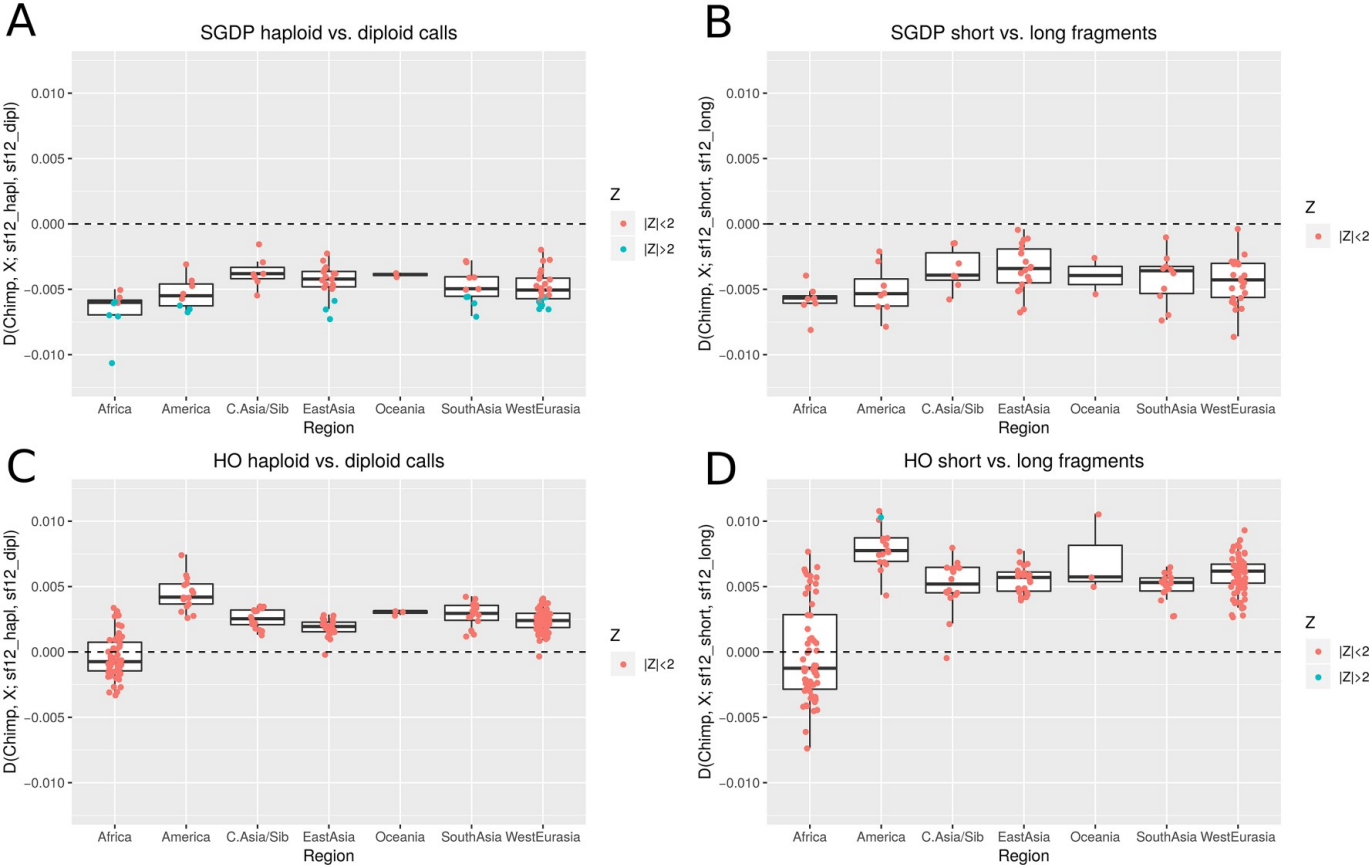
*D* statistics testing the affinity between different modern populations (*X*) and two different treatments of the high coverage individual sf12. The basis for these comparisons are the whole genome sequence data of the SGDP panel (A and B) or SNP array genotype data from the HO panel (C and D). Comparisons are done between pseudo-haploid and diploid calls for sf12 (A and C), and between pseudo-haploid calls from short (35-40 bp) or long (75-80 bp) fragments (B and D). The x axis represents the geographic origin of population *X*.

We use four different versions of genotypes for sf12. First, we compare pseudo-haploid calls (random allele per site with minimum mapping and base quality of 30) to diploid genotype calls (Fig 3A and 3C). This comparison assumes that the diploid calls are less affected by reference bias as slight deviations from a 50/50-ratio at heterozygous sites should be tolerated by a diploid genotype caller but random sampling would be biased towards the reference allele. This is supported by the *D* statistic *D*(*chimp*, *reference*_*genome*; *sf*12_*hapl*, *sf*12_*dipl*) < 0 (*Z* = −7.2), indicating more allele sharing between the reference and the pseudo-haploid calls. For this illustration, we are using diploid genotype calls from *GATK* as we are only looking at the variation at known SNP sites. We note that different calling methods might also introduce other types of technical artefacts and that genotype callers specifically developed for ancient DNA [40–42] are preferable when calling novel variants from ancient DNA data as they incorporate post-mortem damage and other ancient DNA specific properties. Second, we compare randomly sampled reads of different fragment length categories (Fig 3B and 3D) as longer (75-80 bp) fragments should exhibit less reference bias than short (35-40 bp) fragments (see above), which is supported by the *D* statistic *D*(*chimp*, *reference*_*genome*; *sf*12_*short*, *sf*12_*long*) < 0 (*Z* = −5.8), indicating more allele sharing between the reference and pseudo-haploid calls from short fragments.

In general, we observe a deviation from zero in most cases highlighting the effect of reference bias on these statistics (Fig 3). Surprisingly, the directions of this bias differ between the HO data (SNP genotyping array) and the SGDP data (whole genome sequencing), which suggests that different reference data sets are also affected by reference bias at different degrees. Even when investigating the modern populations at only sites that were covered in both data sets, we see differences in the relative heterozygosity for the same individual between the data sets (S3 Fig). The SNP array data (HO) consistently shows lower heterozygosities and a higher count of reference alleles for all individuals which might be a consequence of the different calling algorithms employed for these fundamentally different data types. This represents a potential batch effect which also needs to be considered when merging different reference data sets. Affinities to populations of different geographic origin vary in their sensitivity to reference bias but little general trends are observable. Western Eurasian populations show a strong deviation from 0 in all tests. Some of these individual tests would have achieved nominal significance (assuming a significance threshold of |*Z*| > 2 and no correction for multiple testing). Notably, African populations show the strongest deviation in the short versus long comparison in the SGDP data set while they exhibit almost no bias in the same comparison using the HO data. As the biases do not seem to show a consistent tendency, we cannot directly conclude that recent ancient DNA papers have been systematically biased in some direction. The shifts appear to be dataset and test specific so some results could still be driven by spurious affinities due to reference bias.

**Fig 4:**
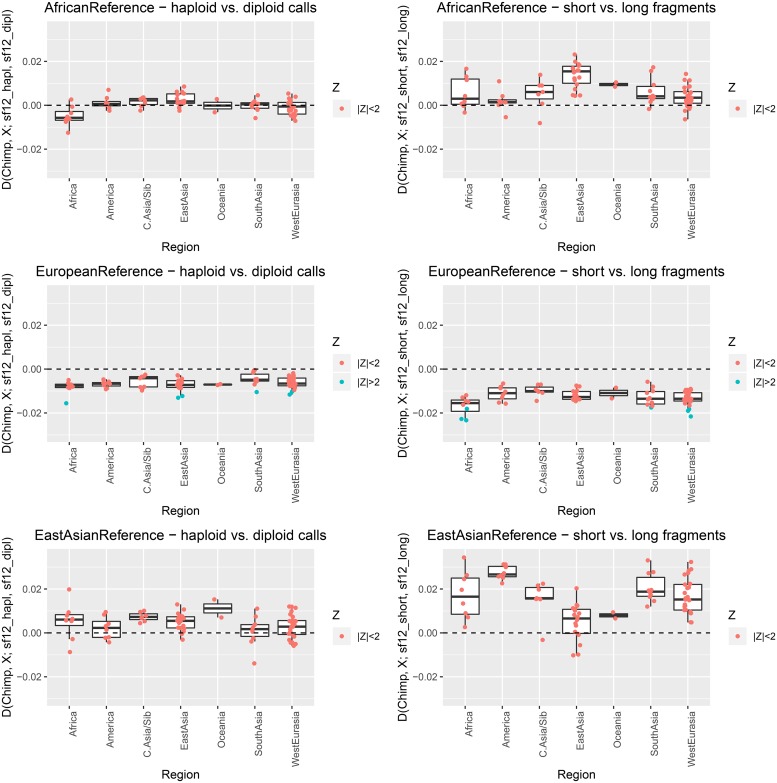
*D* statistics similar to Fig 3 for different parts of the reference genome depending on their geographic origin [44]. The x axis represents the geographic origin of population *X*.

The human reference genome sequence is a mosaic of the genomes of different individuals, and population specific segments might not be well represented in the reference assembly [43]. The geographic origin of the specific segments should have an impact on the population genetic affinities as the reference allele will more likely be found in specific geographic regions. We obtained information on the local ancestry of the human reference genome from [44]. According to this estimate 15.6% of the reference genome can be assigned to African, 5.0% to East Asian and 30.0% to European origin while the origin for 49.4% is uncertain. We re-calculate *D* statistics for the different parts of the genome separately, restricting the analysis to the SGDP data. The impact of reference bias differs between the different ancestries (Fig 4). Generally, reference bias is weakest for reference segments of African origin. Notably, African populations show the strongest deviations from 0 in this case. Sequences mapping to the European segments of the reference show a strong reference bias with slight differences between continental populations. Several tests show nominal significance (|*Z*| > 2) for higher allele sharing of the modern group with the more biased version of sf12. Reference bias at the East Asian segments of the reference genome seems intermediate but the *D* statistics also show large variation and noise which may be due to the only small proportion of the reference genome that could confidently be assigned to an East Asian origin [44].

Finally, we explore whether reference bias can affect estimates of archaic ancestry. We estimate the Neandertal ancestry proportion in sf12 as done by [34]:

$$\begin{array}{c} \alpha =\frac{f_{4}\left(sf12,Mbuti;AltaiNea,Chimp\right)}{f_{4}\left(VindijaNea,Mbuti;AltaiNea,Chimp\right)} \end{array}$$

We use eight different combinations of diploid and pseudo-haploid calls for sf12 as well as the two Neandertals in this statistic (Table 3). The 95% confidence intervals of all estimates overlap but point estimates differ by up to 2.85% when using all pseudo-haploid versus all diploid calls. The African segments of the reference genome yield the lowest point estimates (as low as 0.72%)—none of these estimates are significantly different from 0. These numbers alone would not allow to show the presence of archaic admixture in non-African populations—a pattern that has been confirmed using a range of methods other than *f* statistics during the last decade [12]. These different estimates highlight some of the sensitivities of *f*_4_-ratios not just to the choice of reference populations [45] but also to technical artifacts.

**Table 3:** Percentage of Neandertal ancestry (and standard errors) in sf12 using diploid and pseudo-haploid calls and different subsets of the human reference genome. Parts of the genome of East Asian origin were excluded due to their small total size.

Statistic^$^	Full reference	European Reference	African Reference
$\frac{f_{4}\left(sf12_{h},Mbuti;AltaiNea_{h},Chimp\right)}{f_{4}\left(VindijaNea_{h},Mbuti;AltaiNea_{h},Chimp\right)}$	3.17 ± 0.47	3.73 ± 0.82	1.76 ± 1.01
$\frac{f_{4}\left(sf12_{h},Mbuti;AltaiNea_{d},Chimp\right)}{f_{4}\left(VindijaNea_{h},Mbuti;AltaiNea_{d},Chimp\right)}$	2.51 ± 0.46	2.97 ± 0.81	1.04 ± 0.99
$\frac{f_{4}\left(sf12_{d},Mbuti;AltaiNea_{h},Chimp\right)}{f_{4}\left(VindijaNea_{h},Mbuti;AltaiNea_{h},Chimp\right)}$	3.00 ± 0.44	3.44 ± 0.77	1.38 ± 1.01
$\frac{f_{4}\left(sf12_{d},Mbuti;AltaiNea_{d},Chimp\right)}{f_{4}\left(VindijaNea_{h},Mbuti;AltaiNea_{d},Chimp\right)}$	2.34 ± 0.44	2.71 ± 0.76	0.72 ± 1.01
$\frac{f_{4}\left(sf12_{h},Mbuti;AltaiNea_{h},Chimp\right)}{f_{4}\left(VindijaNea_{d},Mbuti;AltaiNea_{h},Chimp\right)}$	2.98 ± 0.47	3.43 ± 0.83	1.79 ± 1.02
$\frac{f_{4}\left(sf12_{h},Mbuti;AltaiNea_{d},Chimp\right)}{f_{4}\left(VindijaNea_{d},Mbuti;AltaiNea_{d},Chimp\right)}$	2.34 ± 0.46	2.71 ± 0.82	1.15 ± 0.99
$\frac{f_{4}\left(sf12_{d},Mbuti;AltaiNea_{h},Chimp\right)}{f_{4}\left(VindijaNea_{d},Mbuti;AltaiNea_{h},Chimp\right)}$	2.89 ± 0.44	3.18 ± 0.78	1.46 ± 1.02
$\frac{f_{4}\left(sf12_{d},Mbuti;AltaiNea_{d},Chimp\right)}{f_{4}\left(VindijaNea_{d},Mbuti;AltaiNea_{d},Chimp\right)}$	2.19 ± 0.44	2.46 ± 0.77	0.88 ± 1.01

^$^ *d* and *h* denote diploid and pseudo haploid-calls, respectively

### Potential data filtering strategies

After establishing the abundance and potential effect of reference bias, we investigated two simple post-mapping filtering approaches to mitigate reference bias. The two agents involved in the process are the reference genome and the sequence fragments or reads.

First, we modified reads that successfully mapped to a SNP site with a match of the reference allele to carry the alternative allele. These modified reads were re-mapped to the reference genome and they passed the filtering if they still mapped to the same position of the genome with no indels. Second, we prepared a modified version of the reference genome which carried a randomly chosen third base (neither the reference base nor the known alternative allele) at all 1,022,984 sites. A similar approach has been used to study ultra-short fragments in sequence data from archaic hominins [46]. All reads originally mapping to the SNP sites were re-mapped to this modified reference genome, and again only reads that mapped to the same location and without indels passed the filtering. Finally, we used both filters on the same BAM file. All scripts used for filtering can be found at https://bitbucket.org/tguenther/refbias/.

**Fig 5:**
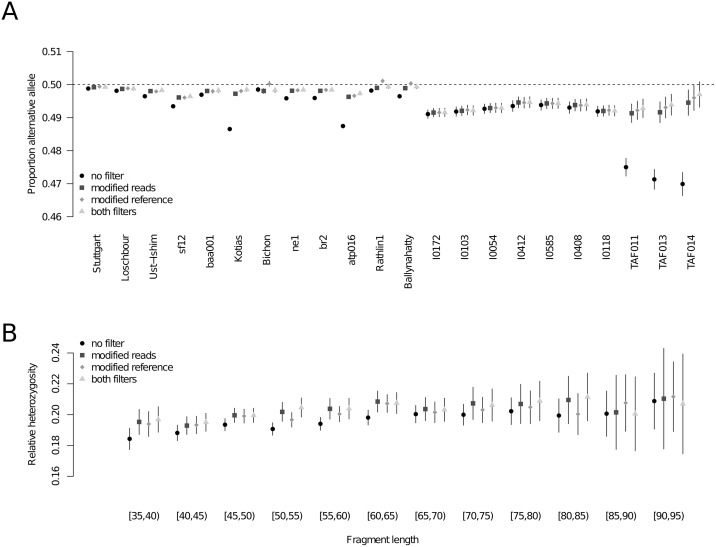
Comparison of different post-mapping filtering strategies for high coverage bam files from anatomically modern humans employing mapping and base quality filters of 30. (A) Average proportion of the alternative allele for the comparison between no additional filters (see also Fig 1), remapping of reads carrying the reference allele modified to carry the alternative allele (modified reads), remapping against a modified reference carrying a third allele at the SNP sites, and both filters together. (B) Influence of filtering on measures of heterozygosity for different fragment sizes in sf12. Error bars indicate two standard errors.

The filtering approaches increase the average proportion of the alternative allele at heterozygous sites (Fig 5A). Mapping to a modified reference genome shows a slightly better improvement than using modified reads, while combining both filters yields the best results in most cases. A small number of samples shows a 50/50-ratio after filtering but most are still significantly below that ratio while three samples even show a slight alternative bias after mapping to the modified reference genome. The limited success of filtering is not surprising as the filtering is only applied to reads that have previously mapped to a single reference genome so the data before filtering does not represent a 50/50-ratio, and removing some reference allele reads cannot completely account for the non-reference reads lost earlier. This is most evident in the samples for which data was not available as raw data including unmapped reads (Table 1) illustrating the importance of sharing all data. Some of these data sets only included mapped reads after running *bwa* [47] with lower maximum edit distance parameters (-n 0.04) than our pipeline which does not leave much room for improvement after filtering. Another possible reason for deviation from a 50/50-ratio at heterozygous sites could be low levels of modern contamination which may lead to a slight over-representation of the reference allele before mapping [33, 42, 48]. Comparing the outcome of the filters to different fragment length categories shows a similar pattern: the bias is decreased but some length categories still display differences in their relative heterozygosity (Fig 5B). We also checked the effect of the filtering on allele sharing with different continental groups by calculating *D*(*chimp*, *X*; *sf*12_*short*, *sf*12_*dualfilter*) which compares the short fragments of sf12 (i.e. high reference bias) with the version after applying both filters (S4 Fig). This is an extreme example to illustrate the effect. The stronger reference bias of the short fragments and the improvement through filtering is indicated by *D*(*chimp*, *reference*_*genome*; *sf*12_*short*, *sf*12_*dualfilter*) < 0 (*Z* = −4.3). In this particular case, *D* statistics tend to be shifted towards the short fragments of sf12 for Americans, Central and East Asians, and Oceanian populations while the tests of Western Eurasian and South Asian populations tend more towards the filtered version of sf12. For the filtered version of sf12, a subsequent analysis of continental ancestry proportions (e.g. using clustering methods [49] or methods based on *f* statistics [50, 51]) could have estimated lower proportions of American, Central and East Asian ancestry, and higher proportions of Western Eurasian and South Asian ancestry. We also compared the filtered version of sf12 to the two treatments with less reference bias, pseudo-haploid calls from long fragments (S5 Fig) and diploid genotype calls (S6 Fig). Consistent with the results shown in Fig 5, there is still some residual reference bias in the filtered data for both comparisons (*D*(*chimp*, *reference*_*genome*; *sf*12_*long*, *sf*12_*dualfilter*) > 0, *Z* = 1.9; *D*(*chimp*, *reference*_*genome*; *sf*12_*diploid*, *sf*12_*dualfilter*) > 0, *Z* = 3.6) but the effect is weaker than in the comparison above (S4 Fig). Furthermore, while the *D* statistics still show skewed results (S5 and S6 Figs), the trends are similar for all continental groups suggesting a reduced impact on downstream analyses.

## Discussion

Systematic biases are problematic in all types of quantitative research, and it is therefore important to be aware of them and alleviate or avoid their effects as much as possible. Different systematic biases in next-generation sequencing data have been investigated before [4, 5, 18, 52], and it is known parameters such as sequencing depth can influence population genomic estimates [53–55]. Differences in sequencing strategies (e.g. read length) and bioinformatic processing have been shown to generate batch effects and dramatically affect downstream analyses [56–59]. Another well known bias in population genetics is ascertainment bias which arises when the studied variants were ascertained in selected populations only, and can substantially impact measurements of heterozygosity and related methods [60]. The research community is aware of these potential issues and they are avoided by filtering strategies, standardizing bioinformatic pipelines, including controls and accounting for systematic biases in downstream analysis.

The common use of randomly sampled alleles and pseudo-haploid data in palaeogenomic research can exacerbate the effect of reference bias compared to diploid genotype calls obtained from medium to high coverage data. We show that reference bias can affect the following types of analyses:

- Heterozygosity and genetic diversity: we observe significantly different estimates of heterozygosity for the same individual depending on what fragment size we are investigating.
- Allele sharing and shared genetic drift: our examples show that reference bias may systematically create spurious signals of allele sharing with certain continental groups which may affect the popular *D* and *f* statistics.
- Ancestry proportions: our analyses illustrate that, because the human reference genome is a mosaic of several different ancestries, genome-wide estimates of archaic ancestry proportions could be slightly affected by reference bias. Additionally, this could impact local inference of archaic introgression tracks.

Our results show that reference bias would be unlikely to bias broad demographic conclusions but it will be more relevant in many future studies focusing on subtle differences between large sample sizes such as weak affinities or small proportions of ancestry. In general, we expect that many other types of analyses that are based on pairwise comparisons between individuals can be affected while methods that work with multiple individuals or groups simultaneously (e.g. PCA or ADMIXTURE) would be less affected.

Mixing different mapping parameters or minimum fragment lengths in the same study should generally be avoided. Additionally, strong differences of fragment size distributions between different individuals may cause spurious affinities due to reference bias. Many estimates from low coverage data are generally noisy, but studies show increasing sample sizes and amounts of data which means that subtle biases become of increasing importance in the future. Notably, the bias for the whole genome (Fig 3) seems less extreme than some of the results for ancestry-specific segments (Fig 4) suggesting that the mosaic nature of the human reference genome may reduce the bias to some degree as different regions will be biased in different directions. In this respect the human reference genome is different from many other species where the reference genome is derived from a single individual which would increase the potential impact of reference bias on population genetic analysis in other systems.

Our analysis indicates a slightly stronger reference bias in SNP capture data compared to whole genome shotgun data. We also observe correlations between samples processed in the same lab or using similar techniques (S1 Table). Different library preparation techniques produce different fragment length distributions since some approaches are directly targeting shorter fragments which will have an impact on mapping. Furthermore, the SNP capture approaches used to generate the data we analyzed uses one bait per allele minimizing reference bias before sequencing. Most whole genome or exome capture approaches, however, are using baits designed from a single individual which should introduce an even stronger pre-mapping bias towards the allele carried by that person [61–64]. Finally, contamination from another person should tend to introduce the major allele which is likely the reference allele in most cases—a process that will also increase reference bias before mapping [33, 42, 48].

Our analysis of the distribution of reference bias across the genome has several repercussions. First, most reads are neutral to changing the allele to its opposing counterpart. This leads to a possible alternative filtering strategy. In cases where a pre-defined set of variants is acceptable, a quality control could be performed on the study level to filter out SNPs which correspond to reads that do not survive this alternative mapping. In our analysis, the sf12 and Ust’-Ishim individuals overlapped in sites with no bias to a marked extent, but this set was scantly larger than the product of the balance fractions for the two individuals suggesting that only a small number of SNPs would have exhibit no bias in multi-individual comparison. There appear to be several individual differences in what SNPs are susceptible to bias which are likely due to preservation, different molecular as well as bioinformatic techniques. The total fraction of SNPs found to be in perfect allelic balance was also markedly higher in the Ust’-Ishim individual, at nearly 90% compared to roughly 60%. However, the total coverage—and consequently resolution—for sf12 was higher, and purely stochastical factors will decrease the proportion of alleles in perfect balance as the number of reads covering each SNP increases.

Another important observation is that reference bias does not occur alone. There is also a weaker, but very clear, signal of alternative allele bias, affecting roughly 4.6% of the total SNPs in the sf12 individual when analyzed using the “virtual allele” method. In addition, both reference and alternative bias can sometimes be very strong on the level of individual SNPs. Even in a dataset with an overall proportion of alternative reads close to 0.5 in heterozygous sites overall, subsets of SNPs might perform very differently, again possibly confusing deeper forms of analysis that do not only consider genome-wide metrics—for example selection scans or analysis of loci involved in certain traits.

We show, that filtering steps can reduce but not completely eliminate reference bias at SNPs after mapping. To fully prevent reference bias, alternative mapping strategies would be needed or filtering strategies would have to be developed for all raw data which is not always published. Our results provide a strong argument for publishing both mapped and unmapped reads in ancient DNA studies. Furthermore, these proposed filters require a pre-defined set of variants used for downstream analysis and are not suitable for calling novel variants from ancient DNA data. The latter, however, will generally be only restricted to high quality and high coverage samples. A recently developed genotype caller for ancient DNA data estimates reference bias from the data and uses the estimate as a parameter for variant calling [42], which seems to work well for samples sequenced to coverages of 15x or higher. One could use the filtering steps tested by us in a similar manner to estimate what proportion of reads in a library are affected by reference bias which could later be used to estimate genotype likelihoods [65, 66]. As reference bias is somewhat predictable and detectable, this offers opportunities to account for it in downstream analyses [7, 67].

Alternative mapping strategies such as mapping against genome graphs [68–70] or multiple reference genomes simultaneously [71] could be able to eliminate reference bias already in the mapping step. These approaches are not broadly established in human genomics yet but their development has huge potential with regard to reference bias. Such approaches could also lead to an increase in the total amount of authentic data that can be obtained from a library while additional post-mapping filters will reduce the amount of data used for downstream analyses (between 2 and 10% in our cases). The first analyses of the Neandertal genome also included a mapping step against the chimpanzee genome to mitigate potential reference bias [44], which should reduce the bias for population affinities but not for other effects such as the presented differences in estimated heterozygosities. In addition to filtering data and standardizing bioinformatic pipelines for all samples used in a study (both published data and newly sequenced), we propose simulations as a potential control. Specific ancient DNA simulation suites [36] provide the opportunity to simulate data exactly matching fragment size and damage patterns of empirical ancient DNA data so one can use them to study if observed patterns may be driven by reference bias alone.

The present study focused mainly on humans but the effect of reference bias extends to other species as well. The slight bias in archaic hominins and the different population affinities depending on the geographic origin of the reference genome illustrate that increasing evolutionary distance can exacerbate reference bias or even cause systematic alternative bias at some sites. This suggests that mapping against a reference genome of a related species (in the absence of a reference genome for the species in focus) may impact downstream analyses as well [1, 2, 19, 20, 44], but the population genetic bias may be weaker as the reference genome employed usually represents an outgroup of equal distance to all individuals in the studied species.

### Conclusion

Our analysis highlights that reference bias is pervasive in ancient DNA data used to study prehistoric populations. While the strength of the effect differs between applications and data set, it is clear that reference bias has the potential to create spurious results in population genomic analyses. Furthermore, even when the overall presence of bias is limited, it is important to assess whether subsets of variants are prone to strong systematic bias, including the possible presence of alternative bias.

We are entering a time where sample sizes in ancient DNA studies reach one hundred and beyond, while the questions focus on more and more detailed patterns and subtle differences. At the same time, sampling starts to involve older remains and remains from more challenging environments—both of which are usually associated with poor preservation and shorter fragments. Therefore it seems crucial to avoid reference bias or other biases such as batch effects or ascertainment biases as much as possible, and to develop and apply computational strategies to mitigate the impact of these issues.

## Materials and methods

### Data sets and bioinformatic processing

We selected medium to high coverage data from 22 different individuals representing data generated by different research groups with different wet lab strategies, covering different geographic regions and time periods (Table 1). For anatomically modern human samples, we tried to use data as raw as possible but some publications only provided the data after mapping and filtering. The general pipeline for these samples was identical to previous studies [25, 72]. Reads were mapped to the 1000 genomes version of the human reference genome hg19 using *bwa* [47] with non-default parameters -l 16500 -n 0.01 -o 2. Subsequently, PCR duplicates and fragments shorter than 35 bp were filtered [73].

We restricted our analysis to a set of known biallelic transversion variants to avoid an effect of post-mortem damage. We selected 107,404 transversions from the Human Origins genotyping array [23, 50] as well as 1,022,984 transversions which were at at least 5% allele frequency in the public data of the Simons Genome Diversity Project (SGDP) [22] and were located in parts of the genome which are uniquely mappable with 35bp reads [33, 35]. To detect reference bias, we are looking at supposedly heterozygous sites where one would expect reads to map in a 50/50-ratio on average if no bias existed. We define a heterozygous site as a SNP for which we observe at least ten reads with between 25 to 75% of those representing the alternative allele. These reads are assessed using *samtools mpileup* version 1.5 [74] employing the -B option to turn off base quality rescaling.

For the high coverage genome of sf12 [25] as well as the high coverage archaic genomes [33–35] we also generated diploid genotype calls similar to the pipeline described in [25]. Briefly, base qualities of the first five base pairs of each read as well as the last five base pairs were set to 2 to avoid residual deamination. *Picard* version 1.118 [75] was used to add read groups to the files followed by indel realignment with *GATK* 3.5.0 [76] based on reference indels identified in phase 1 of the 1000 genomes project [77]. Finally, diploid genotypes were called with *GATK*’s UnifiedGenotyper employing the parameters -stand_call_conf 50.0, -stand_emit_conf 50.0, -mbq 30, -contamination 0.02 and –output_mode EMIT_ALL_SITES using dbSNP version 142 as known SNPs. Genotype calls not flagged as low quality calls at investigated SNP sites were extracted from the VCF files using *vcftools* [78].

When creating the virtual read sets with known perfect heterozygosity in all SNPs, we started out from all reads mapping to SNPs in our marker set, where the read had an original mapping quality of 30, and a base quality of 30 at the SNP base pair. No filter was placed on coverage as this process was fully executed per-read. This joint read set of original and modified reads thus had perfectly balanced allele ratios for all SNPs. The full set was remapped with our pipeline, and SNPs were grouped based on the observed alternative allele fraction among all reads that again mapped to their respective SNPs with mapping quality of at least 30.

### Population genetic tests

In order to investigate the population genetic effect of reference bias, we calculated *D* and *f* statistics [50]. These statistics are based on pairwise allele sharing, so they should be sensitive to spurious allele sharing due to reference bias. *D* statistics were calculated with *popstats* [79], *f*_4_ ratios were calculated *ADMIXTOOLS* [50], and standard errors were calculated employing a weighted block jackknife with a block size of 5 Mbp. We used the chimpanzee reference genome as an outgroup.

## Supporting information

S1 Fig Proportion of alternative alleles for different base quality filters. See Fig 1 for a similar figure for different mapping qualities.
(PDF)

S2 Fig Proportion of alternative alleles for different libraries of the Vindija Neandertal [34]. The three libraries with coverage > 6*X* were selected. Red diamonds represent the mode of the fragment size distribution for each library.
(PDF)

S3 Fig Individual-based comparison of relative heterozygosity between HO and SGDP datasets. Values were calculated only for sites present in both datasets. Relative heterozygosity is shown on the left, total count of reference alleles at all sites on the right.
(PDF)

S4 Fig D statistic comparing allele sharing of sf12 with modern populations for short fragments versus filtered data (after applying both filters to the full data). (PDF)

S5 Fig D statistic comparing allele sharing of sf12 with modern populations for long fragments versus filtered data (after applying both filters to the full data). (PDF)

S6 Fig D statistic comparing allele sharing of sf12 with modern populations for diploid genotype calls versus filtered data (after applying both filters to the full data). (PDF)

S1 Table Pairwise correlations between proportion of reference alleles at heterozygous SNP sites. (XLS)

S1 Data Data shown in figures. (XLS)
