# Supplementary figures and images for: The presence and impact of reference bias on population genomic studies of prehistoric human populations
Source: PLoS Genet. 2019 Jul 26;15(7):e1008302. doi: 10.1371/journal.pgen.1008302 (PMC6685638; doi:10.1371/journal.pgen.1008302)

Minimum BAQ 0

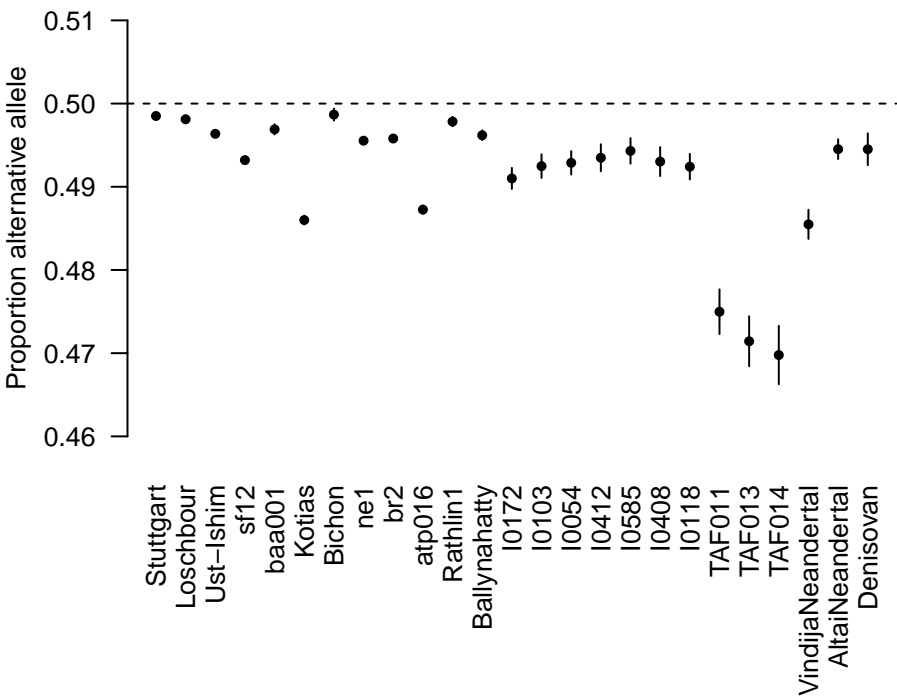

Minimum BAQ 10

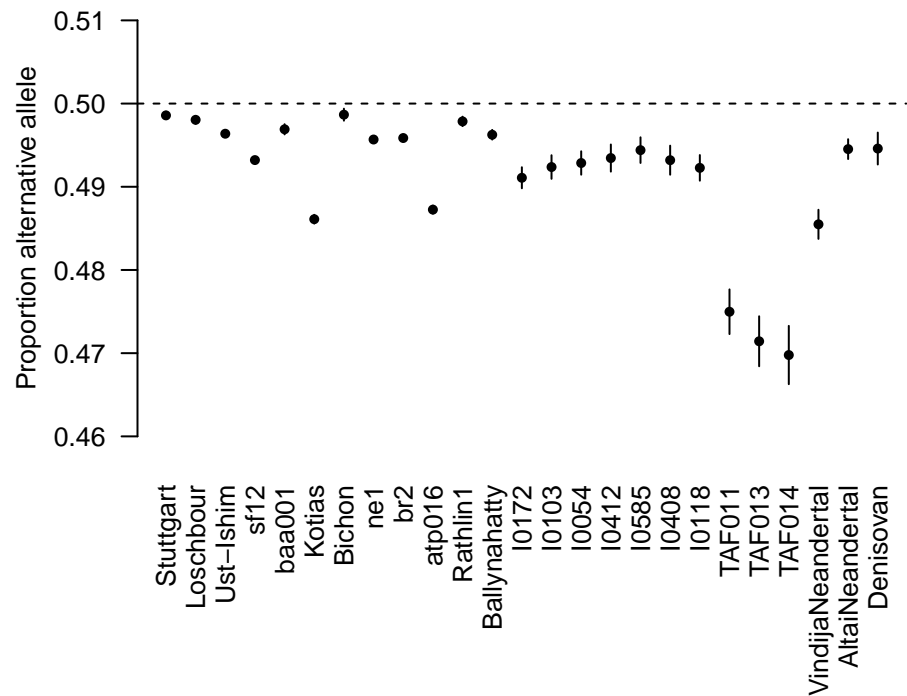

Minimum BAQ 20

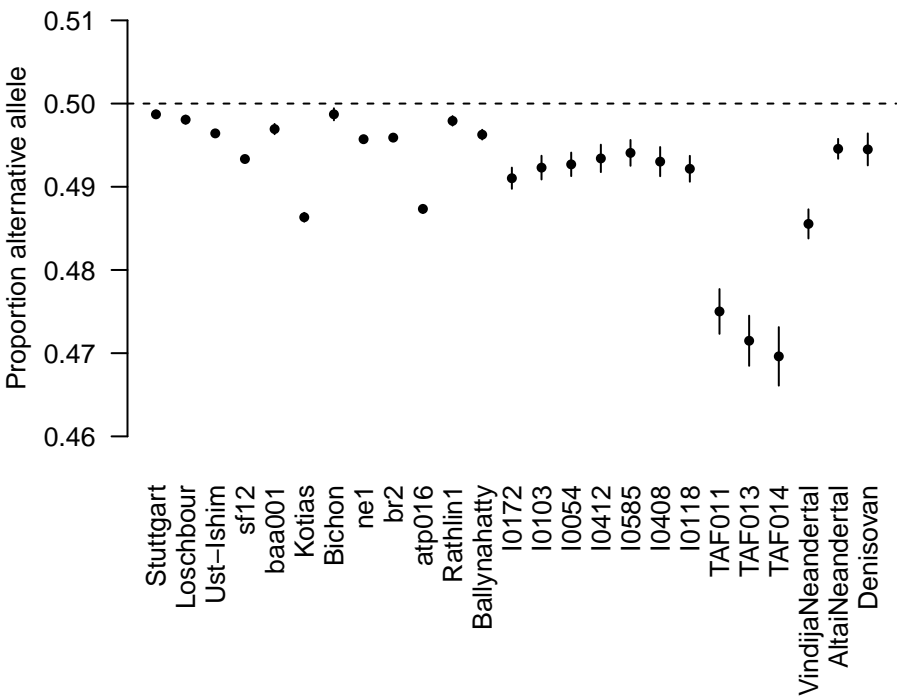

Minimum BAQ 30

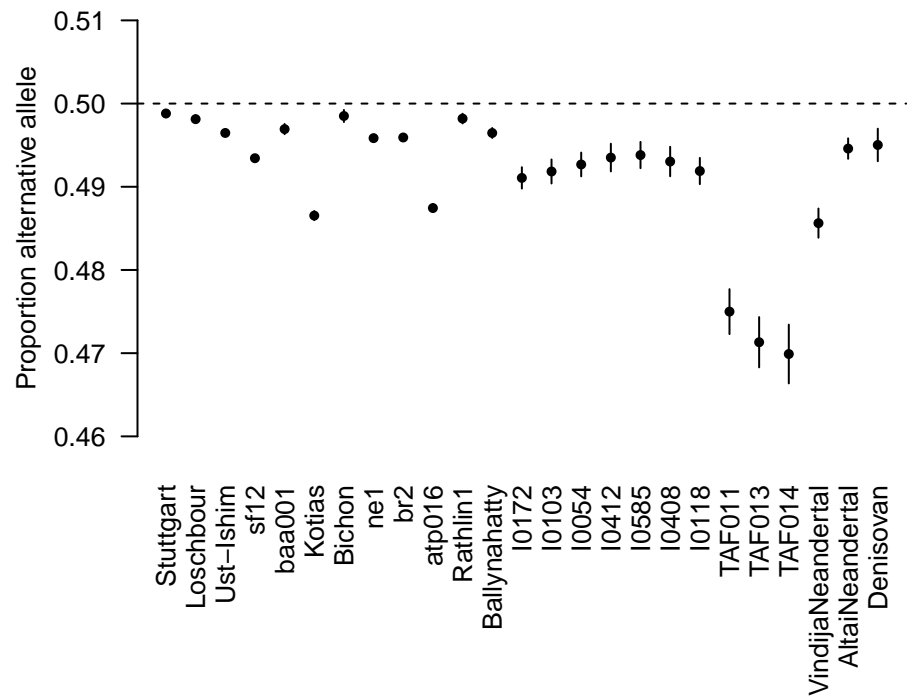

Supplement: S1 Fig — See Fig 1 for a similar figure for different mapping qualities. (PDF) [file pgen.1008302.s001.pdf]

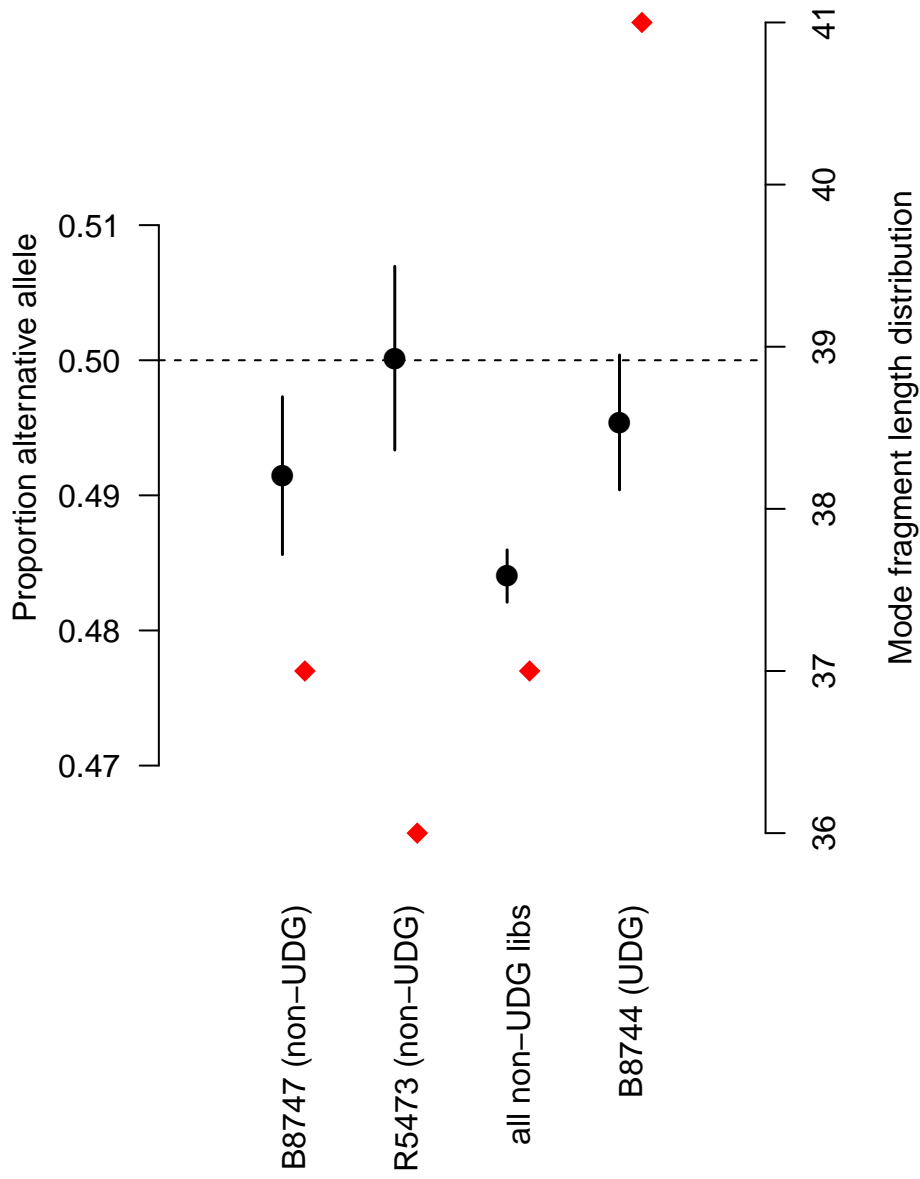

Supplement: S2 Fig — The three libraries with coverage > 6X were selected. Red diamonds represent the mode of the fragment size distribution for each library. (PDF) [file pgen.1008302.s002.pdf]

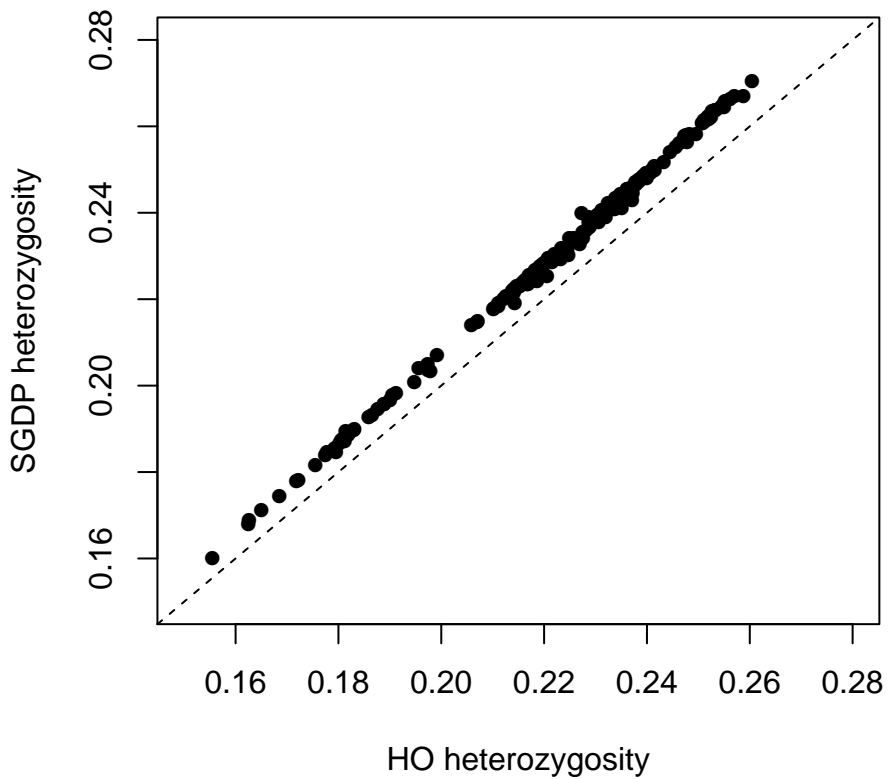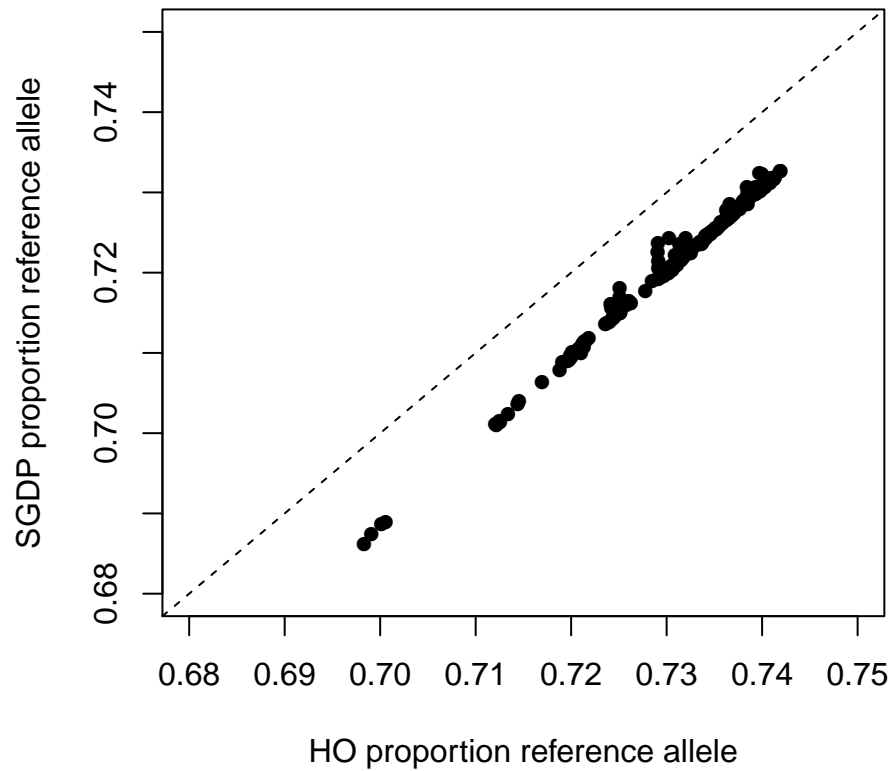

Supplement: S3 Fig — Values were calculated only for sites present in both datasets. Relative heterozygosity is shown on the left, total count of reference alleles at all sites on the right. (PDF) [file pgen.1008302.s003.pdf]

D(Chimp, X; sf12\_short, sf12\_dualfilter)

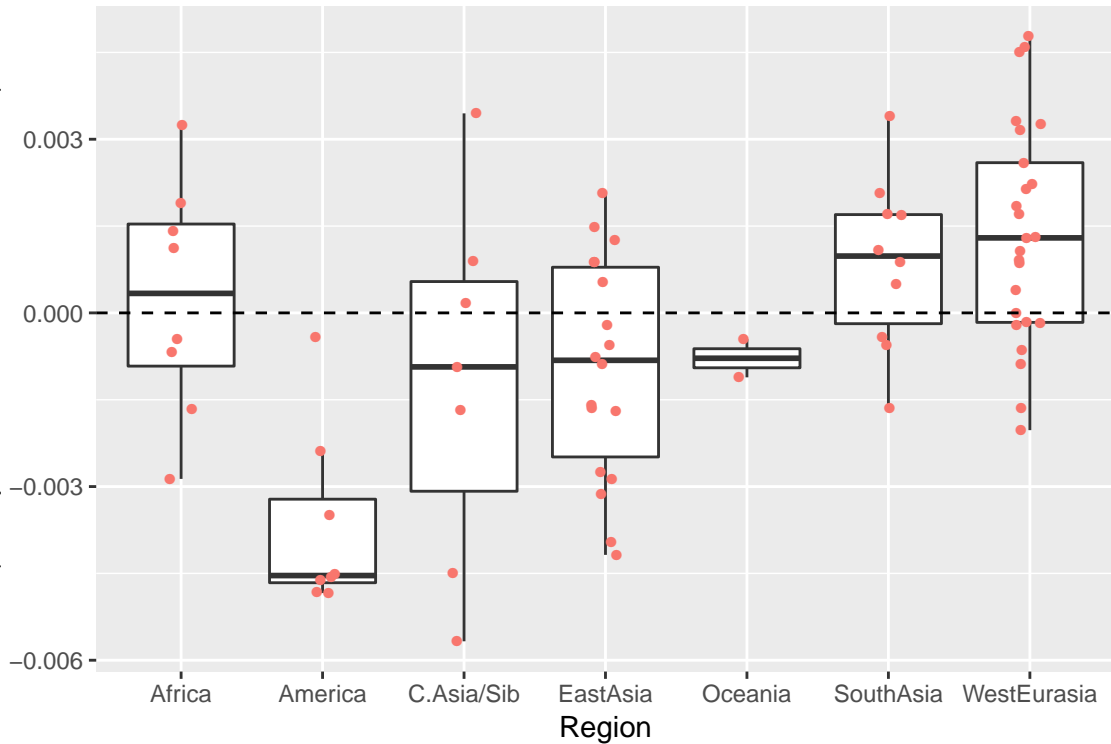

Z

•  $|Z| < 2$

Supplement: S4 Fig — (PDF) [file pgen.1008302.s004.pdf]
