# Supplementary material for: The presence and impact of reference bias on population genomic studies of prehistoric human populations
Source: PLoS Genet. 2019 Jul 26;15(7):e1008302. doi: 10.1371/journal.pgen.1008302 (PMC6685638; doi:10.1371/journal.pgen.1008302)
Supplement: S6 Fig — (PDF) [file pgen.1008302.s006.pdf]

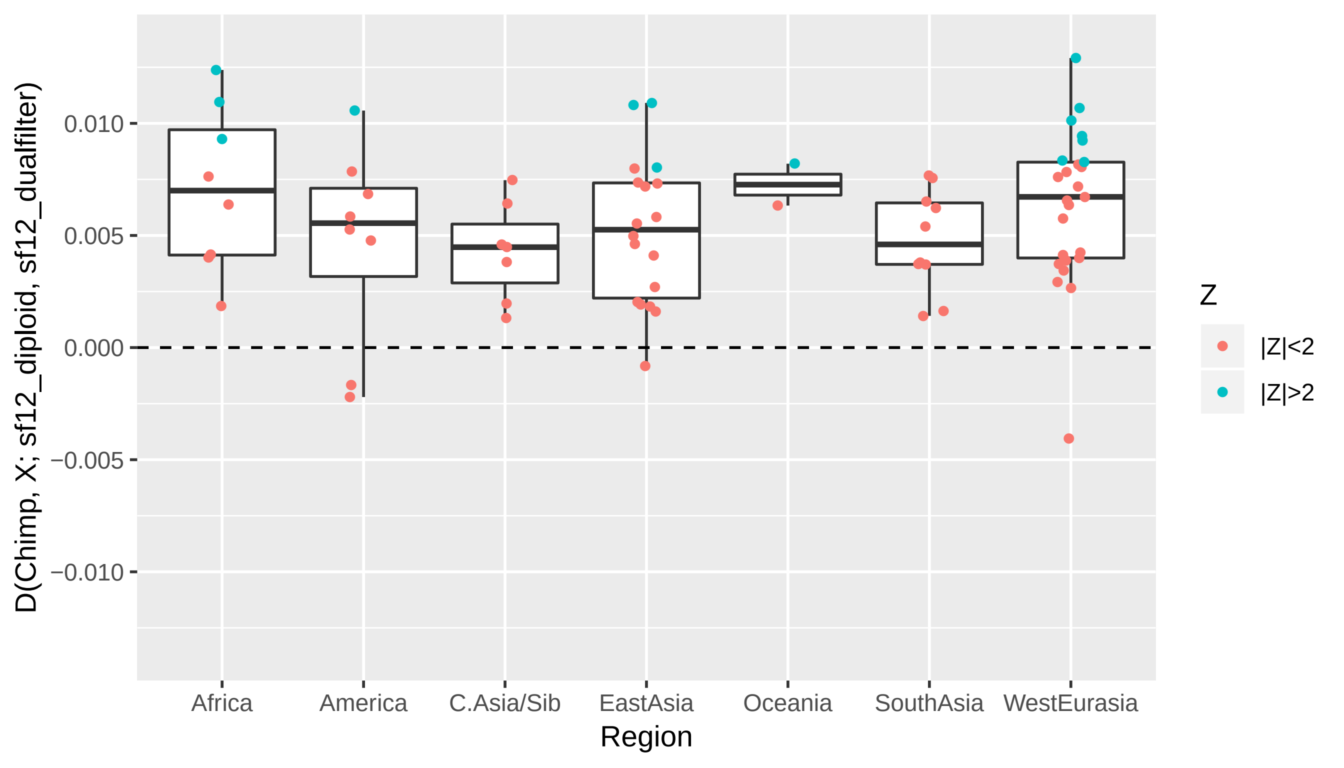
D(Chimp, X; sf12_diploid, sf12_dualfilter)
0.010
0.005
0.000
−0.005
−0.010
Africa
America
C.Asia/Sib
EastAsia
Oceania
SouthAsia
WestEurasia
Region
Z
|Z|<2
|Z|>2
